# Yu-Tao Loo and the development of neuropsychology in China

**DOI:** 10.1007/s13238-017-0376-8

**Published:** 2017-02-23

**Authors:** Yanyan Qian, Wei Chen, Shengjun Wen

**Affiliations:** 1 School of Psychology, Nanjing Normal University, Nanjing, 210097 China; 2 Department of Psychology, Shaoxing University, Shaoxing, 312000 China; 3 Department of Cognitive Neurology, Hertie Institute for Clinical Brain Research, University of Tübingen, 72076 Tübingen, Germany

**Figure 1:**
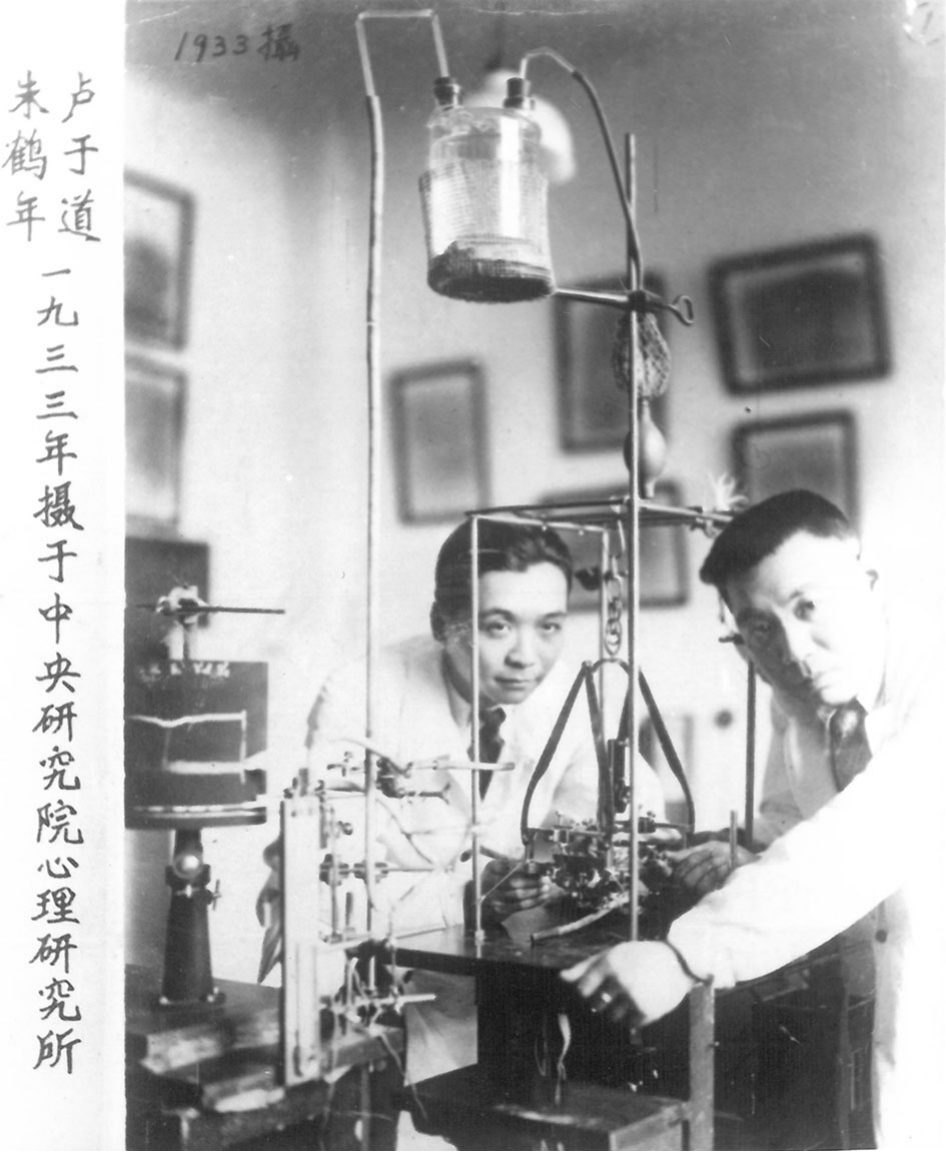
Dr. Yu-tao Loo (from left) with his colleague Dr. Ho-nien Chu (from right) in 1933

Yu-Tao Loo (1906–1985) (Fig. 1) was one of the Chinese neuropsychologists who acted as a pioneer of structure and functions studies of human cerebral cortex (Qian 2013) in China. Loo made great contributions in laying a solid foundation for neuroanatomy and physiological psychology in China. Meanwhile, his academic research broadened the horizons of this field. Besides, as a scientist, he wrote a series of popular-science articles and books to disseminate scientific knowledge. He was also a firm patriot and keen on political construction in China.

Yu-Tao Loo was born on January 9th, 1906 in Yinxian County, Zhejiang Province. In 1921, Loo got accepted into in the Psychology Department of Southeast University (i.e., National Central University), and transferred to Biology Department four years later. After receiving his Bachelor of Science in 1926, Loo won a scholarship sponsored by the Zhejiang Provincial Government to study abroad at the Department of Anatomy of University of Chicago.

Fortunately, Charles Judson Herrick, an illustrious anatomist who has made exceptional contribution to psychobiology, became Loo’s supervisor. In 1930, under Herrick’s guidance, Loo completed his doctoral dissertation *The Forebrain of the Opossum*, *Didelphis Virginiana*. In his study, Loo presented the gross and minute anatomy of the opossum endbrain. The final sentence in his doctoral dissertation revealed the real value and significance of the minute anatomy of the internal structure. That is, “It is hoped that this examination of its internal structure will facilitate the further prosecution of such studies. The generalized character of this brain and the great development of some parts which are reduced or rudimentary in higher mammals also aid in clarifying the general principles of forebrain morphology (Loo 1931)”. The scholars of later generations gave a wonderful comment that his article comprehensively described the anatomy of gross neuroanatomical structures in marsupials (Karlen and Krubitzer 2007). Moreover, Web of Science shows that Loo’s dissertation has been cited for 247 times so far. Even contemporary scientists are developing their scientific researches based on Loo’s exceptional results on brain areas in didelphis, such as Thalamic nuclei (Olokowicz et al. 2008) and claustrum (Hinova-Palova et al. 2008; Mathur 2014). Under Herrick’s recommendation, Loo’s doctoral thesis was published in *The Journal of Comparative Neurology*. Accompanying with this paper, he received a $20,000 grant from the *Rockefeller Brothers Fund*.

In 1930, Loo was recruited as an adjunct professor by the Medical College of National Central University as soon as he returned to China. His responsibilities included giving lectures on experimental anatomy to the university students. Between 1931 and 1939, he worked as a researcher in Psychology Institute of Academia Sinica and took charge of the neuroanatomy laboratory. In 1937, Loo became the main copywriter of *The Chinese Journal of Psychology* where he published a paper *The Functions of the Cerebral Cortex, An Interpretation* that was the only article about physiological psychology in four issues of this journal.

During the Anti-Japanese War, Loo was entrusted by Ren Hong-jun to hold the post as acting director-general of Chinese Science Society, developing social activities in the Rear Areas. In the spring of 1941, Loo moved to Guiyang where he worked as a professor at Xiangya School of Medicine. Between 1941 and 1942, Loo moved to Chongqing, and became an associate professor of Institute of Biology of Chinese Science Society. On July 1st, 1942, Loo served as editor-in-chief for resume publication of Chinese periodical *Science* and published several scientific popular articles himself. Since the autumn of 1942, Loo took a leadership role in Fudan University. He successively held the post of director of Biology Department, assumed the officer of Dean of the School of Science and also served as head of the teaching and research section of Human and Animal Physiology Department. In 1954, Loo established the major of human body and animal physiology with Zongpeng Sun.

In 1950, Loo attended the National Congress of Natural Science Workers in China and became committee member in preparatory office of the founding committee of the Chinese Psychological Society (Jing 2001). The Chinese Academy of Sciences (2004) carried out a nationwide election to summarize the contributions of natural scientists from 1949 to 1950 and ranked the natural scientists according to the significance of their academic contributions. As a result, Loo was nominated in two scientific disciplines in this election. He ranked 50th in experimental biology group and 23rd in psychology group.

After the founding of New China, Loo took an active part in the work of Chinese societies and science popularization. In 1947, Loo was elected as the member of electoral commission of China Zoological Society that had just reestablished. In July 1947, Loo became the first chairman of board in Chinese Society for Anatomical Sciences. In 1950, Loo was one of the 15 principals to organize Chinese Psychological Society. And in May, Loo took part in the creation of Chinese Association of Anthropology with Ting-liang Woo, Chungshee H Liu and so on.

In August 1954, Loo was elected as president of Shanghai Science and Technology Popularization Society. Between November 20th and 23th, 1958, Loo was elected as first vice-chairman of Shanghai Association for Science and Technology and stayed the course for two terms.

In June 1970, Loo and Hsiang-tung Chang jointly presented proposals to set up an institution of brain research in Chinese Academy of Sciences at the Third Session of the Fifth National People’s Congress. The congress adopted their proposal. In 1980, Shanghai Brain Research Institute of Chinese Sciences Academy was established in Shanghai. With their efforts, the brain research in China entered into a new stage.

As Loo said, “his research pattern can be categorized into three directions after returning to China. The first of these is on Chinese brains. The second aspect is on continuing to doing research on Herrick’s comparative neurology. And the third aspect is on detecting nerve cells by means of microscopic chemistry (Loo 1948).”

First, Loo made pioneering contributions in the study of tissue and structure of human brain. In 1929, through studying the development of the cerebral cortex by dissecting fetal brains, Loo wanted to find out a special kind of cells—labile type cells which are chiefly responsible for higher mental activity and knew if there are any cells remaining to have more embryonic potency than others. Loo summarized his research in a paper entitled *On formation of human cerebral cortex ontogenetic study with a discussion on the functions of different cortical layers* (Loo 1929). This paper has rendered him one of the first scientists engaged in structure and function in the central nerve system in China (Xu 2000). In 1931, *Neuroanatomy*, compiled by Loo, became the first textbook on the anatomy of human nervous system in China that systemically introduced the structure and function of human central nervous system.

Building on previous human brain research, Loo compared the human brain of Chinese with that of Negro and White. In 1926, Shellshear Joseph Lexden, a scholar in Hong Kong, believed that Chinese brains were more anthropoid (Shellshear 1926). This formed the basis of Shellshear’s speech that Chinese brain is not as powerful as European at the First International Union of Anthropological and Ethnological Sciences held in London in 1934. This incidence solidified Loo’s decision to explore the difference in the brain between Chinese and Europeans (Loo 1934). During Loo’s stay in the Western Reserve University, Cleveland, Ohio, United States, he observed and collected statistical data on fifty Chinese brains in comparison with fifty Negro and fifty White. Though Loo’s research findings verified Shellshear’s research data, Loo still rebutted Shellshear’s claim with a supporting detail that sulcus does not appear in ontogentically fetal brains. Furthermore, Loo proposed three hypotheses to contradict Shellshear’s previous conclusion that Chinese brains is inferior to Western brains and explained the higher percentage of sulcus lunatus in Chinese brains: (1) visual area may be better developed in Chinese; (2) parietal may be more advanced in Chinese; (3) the longitudinal axis of the brachycephalic brain is shorter in Chinese (Loo 1933).

Second, Loo continued to make outstanding contribution to comparative neurology. Loo performed a systematic comparison in different mammals. From 1932 through 1937, Ho-nien Chu, his fellow classmate collaborated with Loo to study the vasomotor responses of the midbrain with the Horsley-Clarke apparatus in the cat. They discovered a special phenomenon named groaning reaction (Loo and Chu, 1937a, b). It was the first recorded observation of groaning response. Moreover, they imbedded electrode in cat’s different brain regions to perform chronic experiment (Chu 1985). In 1942, as an associate professor of Institute of Biology of Chinese Science Society, Loo completed a book entitled *The Evolution of the Brain* after cumulating experience in dissecting the brain of weasel, civet cat, leopard, panda and so on. This book won Loo the Second Class Prize of National Natural Science (Hu 2014).

In 1941, while at the Institute of Psychology, Academia Sinica, Loo performed an extensive microscopical survey on the septum of adult brains in different mammals (e.g., bat, hedgehog, mole, mouse, rat, rabbit, pangolin, cat and dog) to expose the evolution of mammalian brains (Loo 1941a). Loo identified a group of cells which were constantly consistently present in various different mammal species as the vestigial paraphysis (Loo 1941d). In 1947, Loo reused the brains of mammals that he collected in 1941, and discovered that the strio-amygdaloid complex (except the claustrum) underwent differentiation with the evolution of the mammals (Loo 1941b). In 1957, Loo compared the brains of cats, leopard cats, bears and pandas, through these studies Loo found that pandas belong to bears (Loo, 1957a, 1957b). Based on above research, Loo proposed that the orthogenesis theory may not be valid (Loo 1948).

Third, by focusing on thymus nucleic acid, and nuclear protein, etc. in brain cortex, Loo contributed in the field of neurochemistry. He found that the histochemical pattern of the distribution of the thymo-nucleic acid in the nucleus varied in different cell types (Loo 1936a). In another study on the distribution of thymo-nucleic acid in the normal adult nerve cells, Loo showed that thymo-nucleic acid, the acid part of the nucleo-protein compound, presented both intranuclearly and extranuclearly in a specialized cell type (e.g., the Purkinje cell) (Loo 1936b).

In addition to the contributions mentioned above, Loo analyzed human constitution from the perspective of nervous system. Loo (1941c) supposed that, “human constitution is the physical make-up and their interrelationships of an individual with predisposition to health and disease”. With the financial sponsorship from the Ministry of Education and assistance from Ketan Ma, Nan-hsuan M. Woo and Li Chan (fellows from Department of Animal Science, Institute of Biology, Chinese Academy of Sciences), Loo examined physical and mental health of Chinese adolescents either in physiological or psychological type (Loo 1945).

Finally, Loo contributed to the promotion of popularization of scientific knowledge, and fostered the next generation of young scholars. As one of the founding members of *Science Illustrated*, Loo influenced many young people to pay close attention to science, such as Xueqin Li, a well-known contemporary historian and expert in ancient writing (Li and Zhang 2008). Loo also cultivated additional junior faculty members. For instance, Hsiang-tung Chang learned neuroanatomy from Loo in his sophomore year and entered Anatomy laboratory established by Loo; Fenhan Zhao was influenced by Loo and became a technologist specialized in neurohistology (Zhang 2012). Loo wrote a series of popular science articles and chapters, such as *The Living Body*,* The Introduction to Science*.

Loo is known as a psychologist, a neuroanatomist and a patriotic personage. For Loo, psychology was the first step on the road towards a career in scientific research. Neuroanatomy, to a certain degree, was a stepping stone to psychology, which made him study neuroanatomy. As a patriotic personage, Loo’s rich accumulation had broken forth vastly for 20 years, which contributed tremendously to the development of neuropsychology in China.
